# CFTR is required for the migration of primordial germ cells during zebrafish early embryogenesis

**DOI:** 10.1530/REP-17-0681

**Published:** 2018-06-21

**Authors:** Huijuan Liao, Yan Chen, Yulong Li, Shaolong Xue, Mingfeng Liu, Ziyuan Lin, Yanyan Liu, Hsiao Chang Chan, Xiaohu Zhang, Huaqin Sun

**Affiliations:** 1 SCU-CUHK Joint Laboratory for Reproductive Medicine Key Laboratory of Birth Defects and Related Diseases of Women and Children (Sichuan University), Ministry of Education, Department of Pediatrics, West China Second University Hospital, Sichuan University, Chengdu, People’s Republic of China; 2 The School of Life Science Shandong University, Jinan, Shandong, People’s Republic of China; 3 Epithelial Cell Biology Research Center School of Biomedical Sciences, Faculty of Medicine, The Chinese University of Hong Kong, Hong Kong SAR, People’s Republic of China; 4 Key Laboratory of Bio-Resource and Eco-Environment of Ministry of Education College of Life Science, Sichuan University, Chengdu, Sichuan, People’s Republic of China; 5 Prenatal Diagnosis Center Department of Obstetrics & Gynecologic, Key Laboratory of Birth Defects and Related Diseases of Women and Children (Sichuan University), Ministry of Education, West China Second University Hospital, Sichuan University, Chengdu, People’s Republic of China

## Abstract

Mutations in the cystic fibrosis transmembrane conductance regulator (CFTR) gene affect fertility in both sexes. However, the involvement of CFTR in regulating germ cell development remains largely unknown. Here, we used zebrafish model to investigate the role of CFTR in primordial germ cells (PGCs) development. We generated a *cftr* frameshift mutant zebrafish line using CRISPR/Cas9 technique and investigated the migration of PGCs during early embryo development. Our results showed that loss of Cftr impairs the migration of PGCs from dome stages onward. The migration of PGCs was also perturbed by treatment of CFTRinh-172, a gating-speciﬁc CFTR channel inhibitor. Moreover, defected PGCs migration in *cftr* mutant embryos can be partially rescued by injection of WT but not other channel-defective mutant *cftr* mRNAs. Finally, we observed the elevation of *cxcr4b, cxcl12a, rgs14a* and *ca15b*, key factors involved in zebrafish PGCs migration, in *cftr-*mutant zebrafish embryos. Taken together, the present study revealed an important role of CFTR acting as an ion channel in regulating PGCs migration during early embryogenesis. Defect of which may impair germ cell development through elevation of key factors involved in cell motility and response to chemotactic gradient in PGCs.

## Introduction

Cystic fibrosis transmembrane conductance regulator (CFTR) is a cAMP-activated anion channel belongs to ATP-binding cassette (ABC) transporter superfamily (Gadsby* et al.* 2006). Mutations of CFTR cause cystic fibrosis (CF), the most common lethal congenital disease in Caucasians (Quinton 1999, Riordan 2008). The most well-characterized mutation ΔF508 affects the trafficking and maturation of CFTR. Another common mutation G551D impairs CFTR channel gating and markedly reduces channel opening probability (Welsh & Smith 1993, Hwang & Kirk 2013). Regardless of the underlying mechanisms, the net outcome of these mutations is the diminished ion channel function of CFTR (Welsh & Smith 1993, Hwang & Kirk 2013). The hallmark of CF is the defects in electrolyte and fluid transport that affect multiple organ systems with a multitude of clinical manifestations (Quinton 1999, Riordan 2008). The reproductive tract is one of the major systems being affected by CFTR mutation. Most CF men are infertile due to anatomical abnormalities of the reproductive tract. Besides, CFTR in sperm may be involved in the transport of HCO_3_−, which is important for sperm capacitation, and CFTR mutations with impaired CFTR function may lead to reduced sperm fertilizing capacity and male infertility (Xu* et al.* 2007). Women with CF have anatomically normal reproductive tracts. Nonetheless, subfertility and infertility are still observed in CF women due to other factors such as ovulation failure. CFTR regulates ovarian estrogen biosynthesis by amplifying the FSH-stimulated signal via the nuclear soluble adenylyl cyclase (sAC), defective CFTR-dependent regulation of estrogen production may underlie the ovarian disorders seen in CF and polycystic ovarian syndrome (PCOS) (Chen* et al.* 2012). Despite the importance of CFTR in the reproductive system, little is known about the role of CFTR in germ cell development.

CFTR is expressed in germ cells of various developmental stages. In the testis, CFTR localized in the cytoplasm and plasma membrane of differentiated germ cells. CFTR and sAC are involved in regulating the cAMP-CREB signaling pathway in Sertoli cells, defect of which may result in impaired spermatogenesis and azoospermia (Snouwaert* et al.* 1992, Xu* et al.* 2011). Besides the roles in differentiated germ cells, CFTR also plays an important role in embryo development as an ion channel (Lu* et al.* 2012, 2016). Intriguingly, apart from its ion channel function, CFTR can also serve as a protein interaction hub and modulate the differentiation of embryonic stem cells via its interaction with β-catenin (Liu* et al.* 2017). The demonstrated roles of CFTR in primitive stem cells and germ cells prompted us to investigate the potential involvement of CFTR in regulating the development of the most primitive type of germ cells, the primordial germ cells (PGCs).

PGCs are the primary undifferentiated stem cell type, which are formed in a distinct position from where the gonad develops at an earlier time and actively migrate to the gonadal ridge during early embryogenesis (Weidinger* et al.* 1999, 2002). The migration of PGCs is regulated by both attractive and repulsive guidance cues established by the somatic cells along the migration path (Paksa & Raz 2015). The number of PGCs arrived the gonad is correlated with sex determination and the onset of oogenesis or spermatogenesis (Nikolic* et al.* 2016). Depletion of PGCs in zebraﬁsh favors testis formation. However, the testis development of PGC-depleted gonads appears to be restrained and delayed, suggesting that PGCs number may directly regulate the variability and length of gonadal transformation and testicular differentiation in zebraﬁsh (Tzung* et al.* 2015).

In zebrafish, the PGCs can be readily detected by marker genes and the migration process is completed within the first day of development. Hence, the zebrafish is an excellent *in vivo* model for investigating the migration of PGCs (Sang* et al.* 2008). Here, we used zebrafish model and two PGCs markers* vasa* and *nanos1* to investigate the function of *cftr* in PGCs during embryo development. Our results showed that the localization of PGCs was impaired in *cftr* mutant embryos, suggesting an important role of *cftr* in regulating PGCs migration during early embryo development.

## Materials and methods

### Ethics statement

All experiments in this study were in accordance with the ‘Guide for the Care and Use of Laboratory Animals’ (Eighth Edition, 2011. ILARCLS, National Research Council, Washington, D.C.) and were approved by the Animal Care and Use Committee of West China Second University Hospital, Sichuan University (Approval ID: HXDEYY20131021).

### Zebrafish and embryos

Zebrafish WT embryos from AB strain were used. Embryos were obtained by natural mating and cultured in embryo medium (Westerfield 1993). Staging of the embryos was carried out according to Kimmel *et al.* (Kimmel* et al.* 1995).

### Cas9/gRNA-mediated cftr mutagenesis in zebrafish

The guide RNA and cas9 plasmid pair was kindly provided by Prof. Bo Zhang (Peking University), and the mutagenesis was performed as described by Chang *et al.* and Liu *et al.* (Chang* et al.* 2013, Liu* et al.* 2014). The *cftr* mutant embryos were obtained by mating heterozygous *cftr* mutant fishes. Mutant lines were bred for three generations to minimize any off-target effects from the genome editing, and we studied the F4 generation for PGCs consequences.

### Reagents and constructs

CFTRinh-172 (Catalog No. S7139) was from Selleck. At the beginning of blastula period (2.5 hpf), embryos (30 embryos in a well of six-well plate with 3 mL culture water) were treated with CFTRinh-172 for 2 h and then subject to whole-mount *in situ* hybridization. The following antibodies were used: anti-CFTR (Abcam, ab2784) and anti-β-tubulin (Epitomics, 1879-1 and Zen Bioscience, 200608).

WT CFTR plasmid is kept in our lab, ∆F508, G551D and I556V mutant plasmids of CFTR were constructed using KOD-Plus-Mutagenesis Kit (TOYOBO). Fragments of PGCs marker genes *vasa* (GenBank# NM_131057, primer ‘CGCGGATCCAGATCAGAGTCCCGTTGTGTCTTGC’ and ‘CCGGAATTCCTCTGCCTTCTCCTCCCTCATCGTT’) and *nonos1* (GenBank# NM_131878, primer ‘CCGGAATTCTGGTGGACAAGAACTACTGCTCGGT’ and ‘CGCGGATCCTTCCTCACATTTTTCACTCCATCAC’) were cloned into vector pSPT18 (Roche, DIG RNA Labeling Kit) for antisense RNA probe synthesis.

### RNA and microinjection

Capped mRNAs were synthesized using mMESSAGE mMACHINE Kit (Ambion). Synthetic capped mRNAs were injected into single-cell embryos. Injection dose was an estimated amount received by a single embryo, ~50 pg mRNA were injected into embryos.

### Zebrafish embryo in situ hybridization

Whole-mount *in situ* hybridization (WISH) was carried out as previously described by Thisse *et al*. (Thisse & Thisse 2008) and Sun *et al*. (Sun* et al.* 2010). After cleavage by appropriate restriction enzymes, antisense RNAs for *in situ* hybridization were synthesized using DIG RNA Labeling Kit (SP6/T7) (Roche) and purified by MEGAclear (Ambion). Antisense RNAs probe template of ca15b was amplified from zebrafish genome by primer ‘TCTACATCAACAACTCCAGCAA’ and ‘GAAATTAATACGACTCACTATAGGG AGACCCGTGACAAGTGAAAACCCACAAT’.

### Quantitative real-time RT–PCR (qPCR) analysis and statistics

Total RNA of each sample was prepared with TRIzol (Invitrogen, 15596-018) from pooled 50 embryos and cDNA was synthesized from 1 μg of RNA with PrimeScript RT reagent Kit (Takara, DRR037A). qPCR with three independent biological replicates and three technical replicates was performed with the SYBR Green detection method with 7500 real-time PCR system (Applied Biosystems). The primers used to detect cxcr4b were ‘GCAGGCTTGAAGGAATTCGG’ and ‘ATTGCTGACTGAGAGGTCGC’; rgs14a were ‘TCACCTGTTTGAATTTGAGGCG’ and ‘GAAACCGCCTGAGTCTGACA’; cxcl12a were ‘CTGTCACAGTTGCTCCTGGAT’ and ‘GGCTTGGCGTTGGAAATCG’; ca15b were ‘TCAGGCTCCGTTTCATGGTG’ and ‘TTGGAGACTGTTGAGTGCCG’; β-actin (housekeeping gene) were ‘ATGAGTCTGGCCCATCCATC’ and ‘CCTTTGCCAGTTTCCGCATC’. All detected genes expression was relative to β-actin. Quantitative data show mean + s.d. The comparative CT (cycle threshold) method (also known as ΔΔCT method) was used to analyze the data. Statistical significance is defined as *P* < 0.05(*), *P* < 0.01(**), *P* < 0.001(***).

## Results

### Ectopic localization of PGCs in cftr-mutant zebrafish embryos

**Figure 1:**
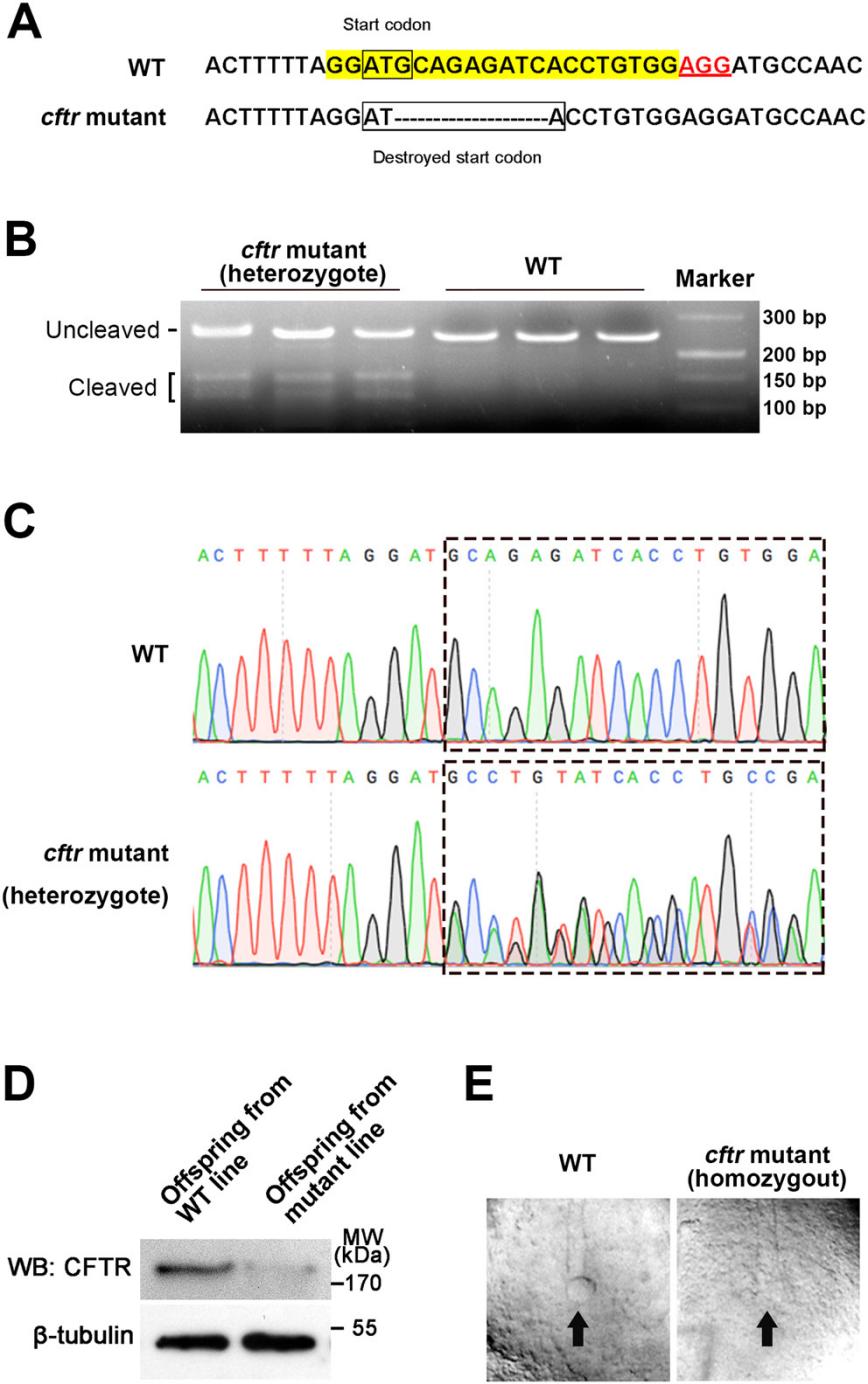
Targeted indel mutation induced by engineered Cas9/gRNA at the *cftr* gene in zebrafish. (A) The target sites highlighted by yellow and the PAM sequence marked by red underlined text. Deletions of *cftr* mutant are shown as dashes. Boxes show the start codon of WT and destroyed start codon of *cftr* mutant. (B) Gel shows T7E1 digestion of PCR products amplified from adult tail genomic DNA of F1 heterozygous generation. The uncleaved and cleaved PCR products are indicated. After digestion with T7E1, the cleaved PCR product of the adult tail represents the fragments containing the mutation. (C) Sequencing results show that F1 heterozygous generation fish carrying* cftr* mutant produces overlapping peaks marked by dashed box. (D) Western blot assay indicates the significant reduced Cftr protein level in offspring embryos from mutant line. (E) The got genotyped homozygous *cftr* mutant embryo also demonstrated the absent of Kupffer’s vesicle (KV) lumen (pointed by arrow) at 8-somite stage.

Similar to the expression profile in mouse embryos, *cftr* was ubiquitously expressed in zebrafish embryos (Sun* et al.* 2018). To investigate the potential involvement of Cftr in PGCs development, we inserted mutations near the start codon of Cftr using CRISPR/Cas9 system (Fig. 1). After screening Cas9/gRNA-injected zebrafish, we identified a heterozygous line that carried a 9-bp deletion (Fig. 1A), which was predicted to delete the start codon of Cftr (Fig. 1A and Supplementary Fig. 1, see section on supplementary data given at the end of this article). We obtained heterozygous male and female fishes by back-crossing the founder with WT AB strain. The homozygous* cftr*-mutant fishes were obtained by crossing the heterozygous lines and screening the offspring. Consistent with previous report (Navis* et al.* 2013), our *cftr* homozygous mutant embryos also demonstrated the absence of Kupffer’s vesicle (KV) lumen at 8-somite stage (Fig. 1E). We also found that a large percentage of homozygous *cftr*-mutant larvae was lost beginning around 10 dpf (Navis & Bagnat 2015). Similar to our previous work (Sun* et al.* 2018), we used heterozygous *cftr* mutant line instead in this study, and the offspring of the heterozygous crosses (mutant line) could be WT (25%), heterozygous *cftr*+/− (50%) and homozygous *cftr*−/− mutants (25%).

**Figure 2:**
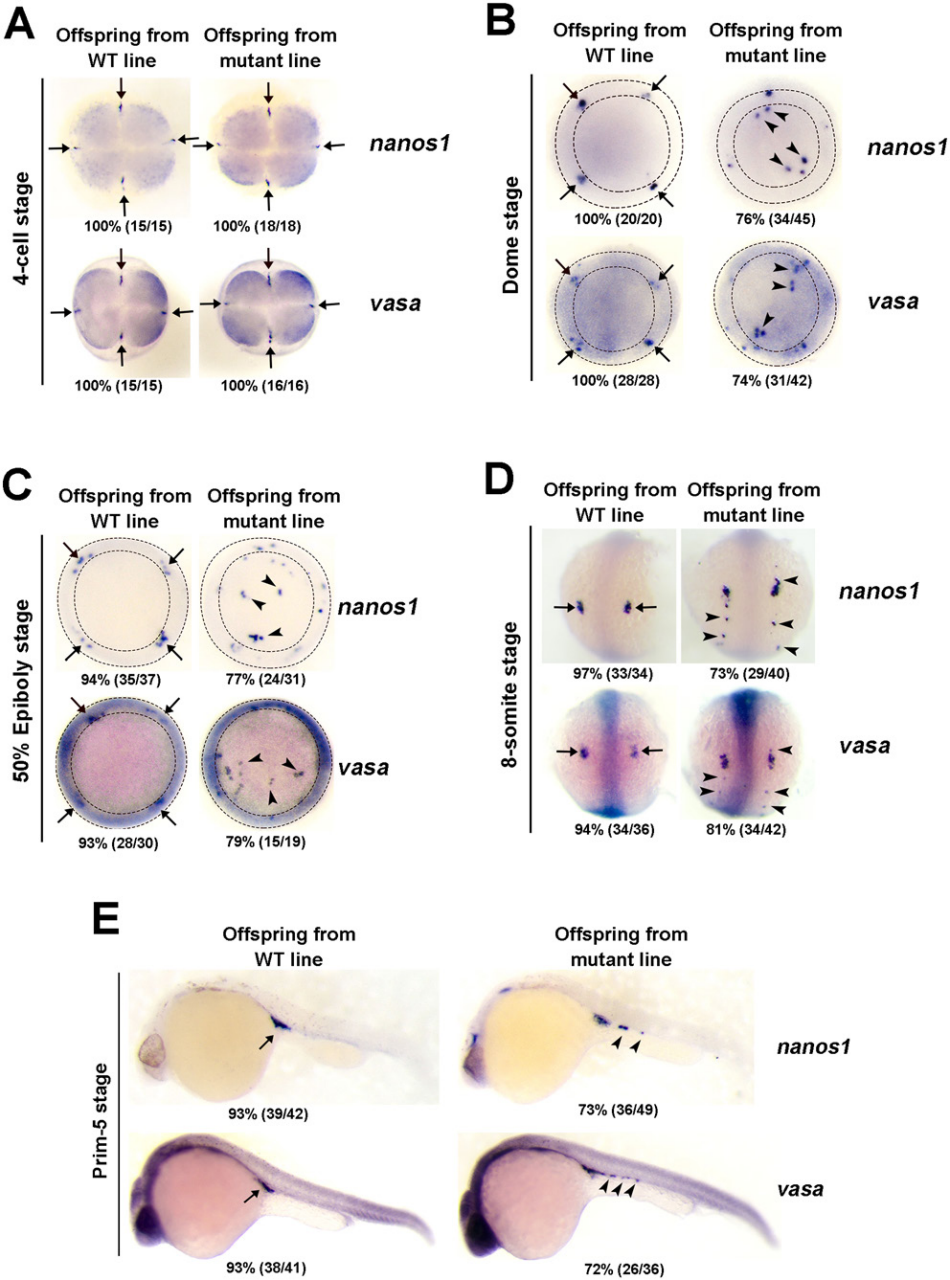
*cftr* mutant induces *nanos1*/*vasa*-marked PGCs disorder in early zebrafish embryo. Analysis of localization of *nanos1*/*vasa* positive cells in offspring embryos from WT and mutant line by WISH at 4-cell stage (A), Dome stage (B), 50% Epiboly stage (C), 8-somite stage (D) and Prim-5 stage (E). Embryo orientations: 4-cell, Dome stage and 50% Epiboly stage, top view; 8-somite, dorsal view with anterior oriented at the top; Prim-5 stage, lateral views with anterior oriented toward the left. Arrows show the normal location of PGCs, arrowheads demonstrate the aberrant position of PGCs in *cftr* mutant. Region between two dotted circles on embryo shows normal location of PGCs. The numbers indicated in each picture are the number (left) of affected embryos with phenotype similar to what is shown in the picture and the total number (right) of observed embryos. The same number labeling was used thereafter.

Next, we compared the PGCs manifestation in offspring embryos from WT and *cftr* mutant line. PGCs are marked by *nanos1* and *vasa* at 4-cell, Dome, 50% epiboly, 8-somite and Prim-5 stage (Raz 2003). Therefore, we examined the development of PGCs by detecting these two marker genes through WISH. In offspring embryos from WT line, PGCs were found in four cell clusters at 4-cell, Dome and 50% epiboly stage (Fig. 2A, B and C). In offspring embryos from mutant line, PGCs were initially observed in four cell clusters at 4-cell stage (Fig. 2A), suggesting that the lineage specification of PGCs was not altered. Intriguingly, when PGCs enter the motile phase at Dome and 50% epiboly stage, some PGCs were dispersed from the clusters, leading to ectopic localization of PGCs in 74–79% of offspring embryos from mutant line (Fig. 2B and C). The ectopic localization of PGCs in >72% of the offspring embryos from mutant line continued at 8-somite stage and Prim-5 stage, where some PGCs failed to migrate to the cell front and reach the genital ridges (Fig. 2D and E and Supplementary Table 1). These results suggested that Cftr is involved in regulating the migration of PGCs.

To verify the corresponding relation between PGCs phenotype and *cftr-*mutant genotype, we chose offspring embryos from mutant line with absent KV lumen at 8-somite stage, and then detected *vasa* expression by WISH, finally performed sequencing to investigate the genotype. Results indicated that embryos with absent KV lumen and disordered PGCs carried the *cftr* homozygous mutant (Supplementary Fig. 1). Interestingly, compared with WT fishes, more male adults were found in *cftr* homozygous mutant fishes even if breeding with low density (Supplementary Fig. 2), suggesting the depletion of PGCs in *cftr* homozygous mutant. These results were consistent with Tzung* et al.’s* discription (Tzung* et al.* 2015); they found that early depletion of PGCs or reduced PGCs number in zebraﬁsh promotes testis formation and increases the percentage of male fishes.

### PGCs migration requires the ion channel function of CFTR

**Figure 3:**
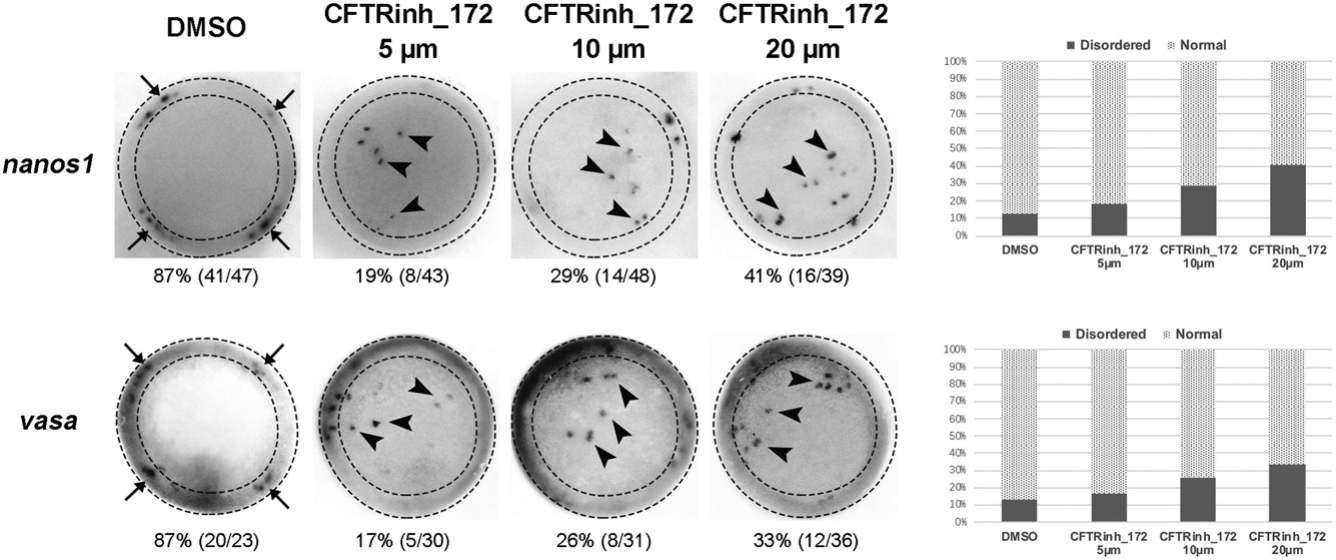
CFTR inhibitor CFTR_inh172 leads to PGCs disorder in early zebrafish embryo. Analysis of localization of *nanos1*/*vasa* positive cells in embryos by WISH at 50% Epiboly stage with top view.

Emerging evidence suggests CFTR can possess both ion channel function and non-ion channel function (Li & Naren 2011). To validate the results from *cftr-*mutant line and to examine the involvement of ion channel function of Cftr in PGCs migration, we treated the WT zebrafish embryos with CFTRinh-172, a potent and speciﬁc inhibitor of the CFTR channel that was identiﬁed by high-throughput screening (Ma* et al.* 2002, Taddei* et al.* 2004). CFTRinh-172 was added at 2.5 hpf for 2 h and embryos were collected at 50%-epiboly stage. PGCs were identified by WISH for *nanos1* and *vasa* as in previous experiment. The results showed that CFTRinh-172 dose dependently (5–20 μM) distorted PGCs migration (Fig. 3). At 10 μM, a dose widely used to inhibit CFTR ion channel activity (Li* et al.* 2004), CFTRinh-172 distorted the migration of PGCs in 26–29% of embryos (Fig. 3).

**Figure 4:**
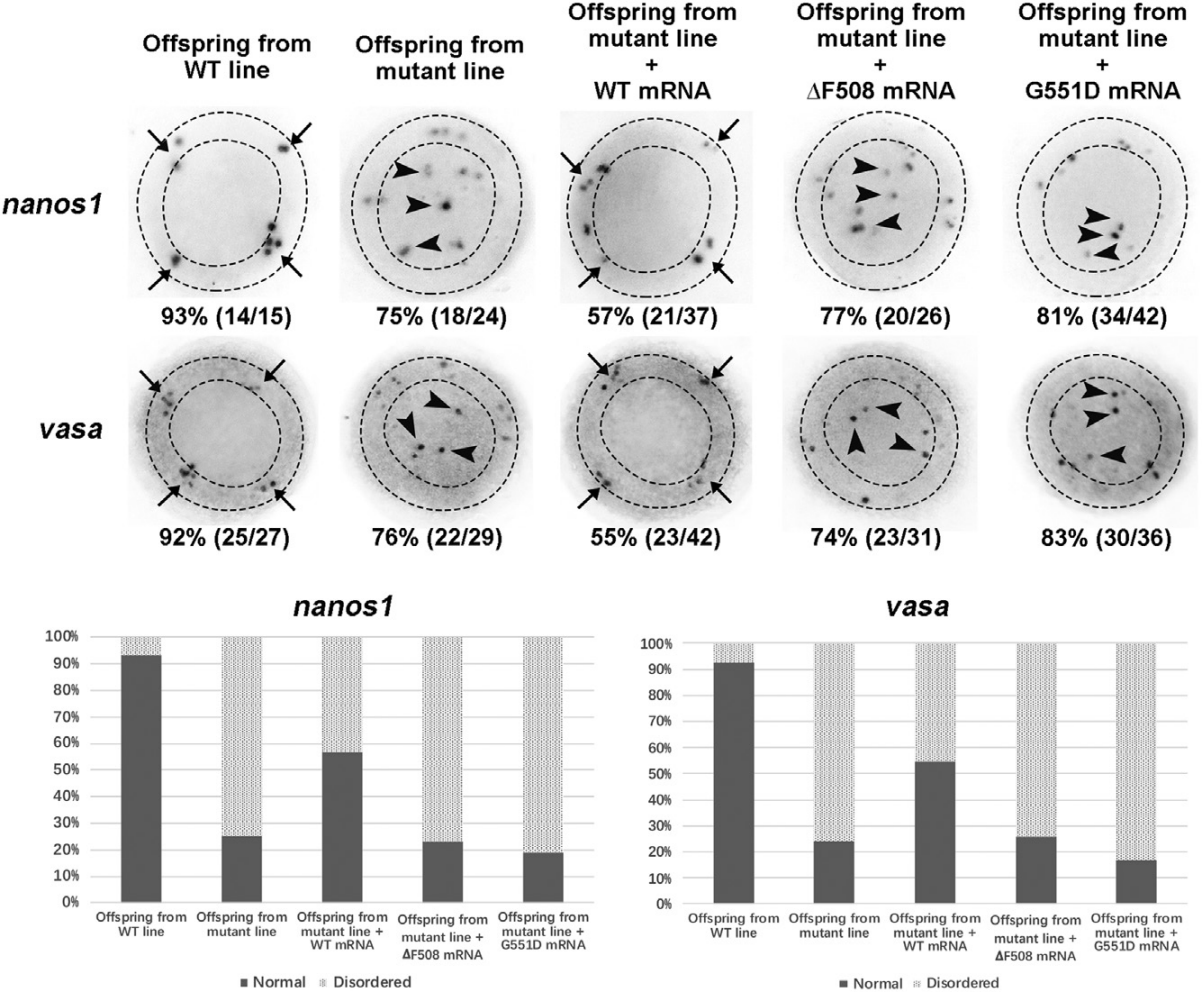
CFTR mutants with loss of ion channel function fail to recover PGCs development. Analysis of localization of *nanos1*/*vasa* positive cells in embryos by WISH at 50% Epiboly stage with top view.

To further validate the involvement of Cftr ion channel function, we sought to restore the migration of PGCs by injecting WT or channel-defective (∆F508 and G551D) human *CFTR* mRNAs into the offspring embryos from mutant line. ΔF508 mutation results in protein misfolding and retention in endoplasmic reticulum (ER) and negligible amount of the mutant CFTR reaching the plasma membrane, leading to impaired ion channel function (Amaral 2004). G551D is a well-known mutation causing gating defect in CFTR channel function (Welsh & Smith 1993, Hwang & Kirk 2013). The results showed that WT *CFTR* mRNA markedly restored the migration of PGCs in offspring embryos from mutant line at 50% epiboly stage. Notably, both of the channel-defective mutants, ΔF508 and G551D, failed to restore the localization of ectopically localized PGCs (Fig. 4 and Supplementary Table 2). Taken together, the results indicated that the involvement of Cftr in PGCs migration requires its ion channel activity.

### Elevation of cell motility factors in offspring embryos from mutant line

**Figure 5:**
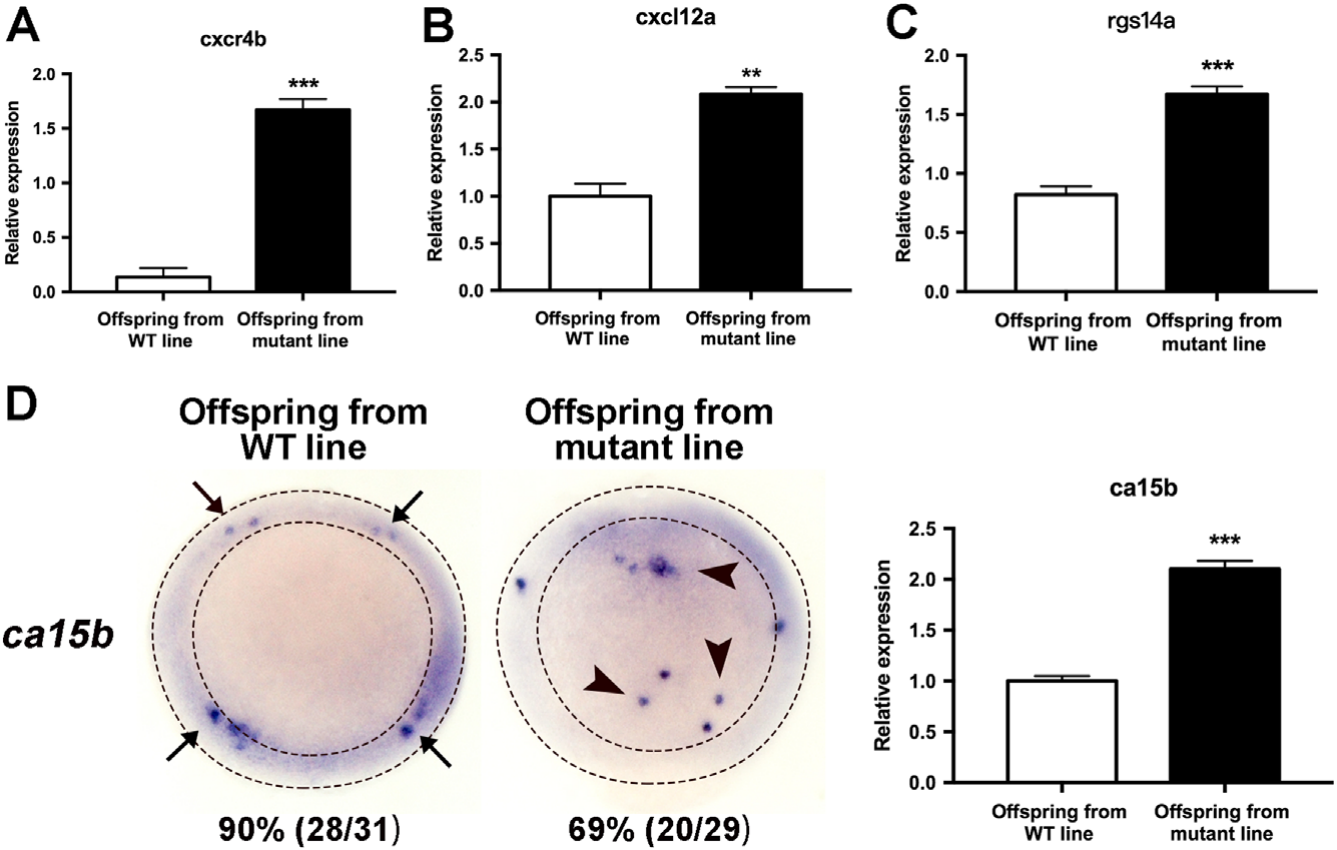
Aberrant expression of key factors (A: cxcr4b; B: cxcl12a; C: rgs14a; D: ca15b) is detected in offspring embryos from mutant line. 50%-epiboly stage embryos were used in these assays. All of the factors were detected by qPCR; ca15b was also detected by WISH.

To decipher the molecular mechanisms underlying the regulation of PGCs migration by Cftr, we analyzed the expression of *cxcr4b, cxcl12a, rgs14a* and *ca15b*,* key* factors that account for the PGCs migration from dome stage to 8-somite stage (Paksa & Raz 2015), by RT-PCR (Fig. 5 and Supplementary Table 3). Cxcr4b is a chemokine receptor (Doitsidou* et al.* 2002) and distributed evenly around the cell perimeter along the PGCs migration route (Meyen* et al.* 2015), initiating an intracellular signaling cascade that biases the formation of protrusions for promoting PGCs migration. Chemokine Cxcl12a is a key attractant for mouse and zebraﬁsh PGCs, which functions upon binding its receptor Cxcr4b in directing the cells to their target (Doitsidou* et al.* 2002, Molyneaux* et al.* 2003). The tight control over the spatiotemporal distribution of Cxcl12a in the embryo ensures an efﬁcient PGC arrival at the target via a reproducible path while keeping the cells away from distant domains where the same chemokine is expressed (Mahabaleshwar* et al.* 2012). Rgs14a, the regulator of G-protein signaling 14a, is expressed in early PGCs germplasm (Hartwig* et al.* 2014), which participates in controlling the timing of PGCs migration initiation. Overexpressing Rgs14a in PGCs induces the formation of protrusions in all directions and the reduction of motility relative to neighboring somatic cells (Hartwig* et al.* 2014). Ca15b (carbonic anhydrase 15b), an enzyme that is expressed specifically in the PGCs, plays an important role in establishment of polar pH distribution for guided PGCs migration (Paksa & Raz 2015, Tarbashevich* et al.* 2015). Our results showed that the expression of *cxcr4b, cxcl12a, rgs14a* and *ca15b* was significantly elevated in offspring embryos from mutant line (Fig. 5). Taken together, these results suggest that Cftr regulates PGCs migration through modulating the expression of factors involved in cell motility and response to chemotactic gradient in PGCs.

## Discussion

CFTR is a well-known anion channel, and CFTR mutations are associated with CF and affect spermatogenesis in males (Xu* et al.* 2011). Besides the differentiated germ cells, the ion channel function of CFTR also plays an important role in embryo development (Lu* et al.* 2012, 2016). The demonstrated role of CFTR in primitive stem cells and germ cells prompted us to investigate the potential involvement of CFTR in regulating the development of the most primitive type of germ cells, the PGCs, in early embryogenesis.

Because of close relevance to mammals, zebrafish is a well-known model for uncovering molecular mechanism regulating PGCs development. To determine the role of *cftr* defects in PGCs development, Cas9/gRNA system was used to establish zebrafish *cftr* mutants. To minimize any off-target effects from the genome editing, mutant zebrafish lines were bred for three generations, and the F4 generation was used in this study. Nevertheless, a large percentage of homozygous *cftr*-mutant larvae was lost beginning around 10 dpf (Navis & Bagnat 2015); furthermore, fertility rate of adult homozygous *cftr*−/− fish was very low, which brought difficulty to experiment. Therefore, we used heterozygous *cftr* mutant line instead in this study, similar to our previous work (Sun* et al.* 2018).

Results showed that *cftr* mutants, both heterozygous *cftr*+/− and homozygous *cftr*−/−, led to ectopic locations of PGCs at different stages of zebrafish embryo development. Importantly, based on genotype result, all embryos carrying *cftr*+/− or *cftr*−/− mutant showed ectopic PGCs localization, suggesting the important and penetrant role of *cftr* in PGCs development. Since offspring embryos from mutant line with mixed genotypes have demonstrated significant change of PGCs and expression of key factors, which can reveal the key role of *cftr* in PGCs already, we did not persist in using homozygous *cftr*−/− fishes.

Consistent with the previous report (Navis* et al.* 2013), our *cftr* homozygous mutant embryo also demonstrated the absence of KV lumen at 8-somite stage. In fact, except for 25% of offspring with absent KV, about 50% offspring embryos from mutant line showed reduced size of KV, which consistent with Roxo-Rosa *et al.*’s description (Roxo-Rosa* et al.* 2015), suggesting that these embryos are most likely the heterozygous *cftr*+/− mutants. Roxo-Rosa *et al.* found that injection of *cftr* AUG-morpholino to knock down Cftr expression severely impaired the lumen expansion of the KV, efficiently phenocopying *cftr* mutants. Furthermore, Navis *et al.* (Navis* et al.* 2013) also showed that their *cftr* ^pd1048^ mutant, maybe a hypomorphic allele, demonstrated the severely reduced KV in size. Taken together, *cftr* plays an important role in controlling lumen expansion and function of KV in zebrafish.

Most interestingly, we found that male adult fishes were dominant in the *cftr* homozygous mutant fishes. This result is consistent with Tzung* et al.’s* report (Tzung* et al.* 2015), they find that the PGCs number is the key factor that control sex determination in zebrafish. Reduced PGCs will promote testis development and increase the proportion of male fishes in the offspring. In *cftr* mutant zebrafish, the number of PGCs that reached the genital ridges was reduced markedly, which resulted in increased male fishes in the *cftr* homozygous mutant offspring.

CFTR is a well-known anion channel, so we asked that whether PGCs migration was regulated by CFTR channel function. Firstly, we used CFTRinh-172, a CFTR channel gating inhibitor, to treat WT embryos, and found that CFTRinh-172 induced abnormal PGCs migration. Next, to get strong evidence of CFTR channel function on PGCs development, we injected mRNAs coding CFTR mutants with gating defect, including ∆F508 and G551D, into offspring embryos from mutant line, respectively. Results showed that these two mutants, compared to WT mRNAs, failed to rescue the distorted migration of PGCs induced by *cftr* mutant. In brief, these results indicate the essential role of CFTR channel function in PGCs migration in early embryos.

Finally, we investigated the expression of key factors that control PGCs migration in offspring embryos from mutant line to understand the molecular mechanism of *cftr* mutant regulating PGCs migration (Paksa & Raz 2015). These factors, including *cxcr4b, cxcl12a, rgs14a* and *ca15b,* control PGCs migration at 50% epiboly stage. Results showed that the expression of all detected factors was increased significantly in *cftr* mutant embryos. These results indicate that CFTR defects have impact on the expression of chemokines.

According to Navis *et al.*'s description (Navis* et al.* 2013) and our previous study, CFTR is expressed ubiquitously during early embryogenesis (Sun* et al.* 2018), suggesting that CFTR may regulate PGCs migration by effect on both neighboring somatic cells and PGCs themselves. Our results show that genes both expressed specifically in PGCs, including *rgs14a* and *ca15b,* and somatic cells, including *cxcr4b* and *cxcl12a,* detected in this work are significantly elevated in offspring embryos from mutant line, suggesting that CFTR sustains PGCs migration through regulating key factors distributed at embryo widely. According to literature work, the precise molecular cascade linking *cxcr4b, cxcl12a, rgs14a* and *ca15b* function is unknown so far (Tarbashevich* et al.* 2015), further investigation is needed in future work.

## Supplementary Material

Supporting Figure 1

Supporting Figure 2

Supporting Table 1

Supporting Table 2

Supporting Table 3

## Declaration of interest

The authors declare that there is no conflict of interest that could be perceived as prejudicing the impartiality of the research reported.

## Funding

This research was supported by grants from National Natural Science Foundation of China (81200339), China Postdoctoral Science Foundation (20110491723), Young Teacher foundation of Sichuan University (2011SCU11040), National Basic Research Program of China (2012CB944903) and National 973 project (2013CB967404).

## Author contribution statement

H S, X Z and H C C conceived and designed the experiments; H L, Y C, Z L, S X and M L performed the experiments; H S, X Z, Y L and H C C analyzed the data; H S, X Z and H C C wrote the paper.
