# Supplementary figures and images for: CFTR is required for the migration of primordial germ cells during zebrafish early embryogenesis
Source: Reproduction. 2018 Jun 21;156(3):261–8. doi: 10.1530/REP-17-0681 (PMC6106808; doi:10.1530/REP-17-0681)

## WT with normal KV

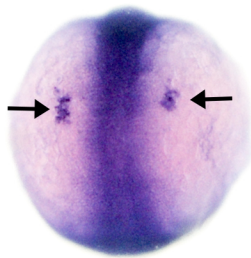

94% (29/31)

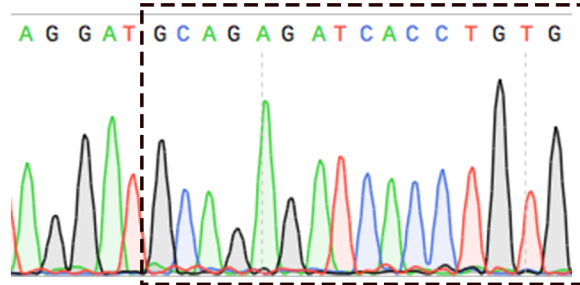

## *cfr* mutant with disappeared KV

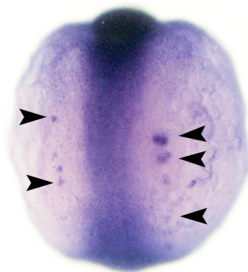

26% (16/61)

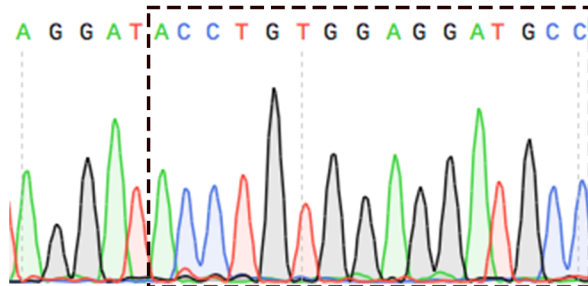

Supplement: Supporting Figure 1 [file rep-156-261-s001.pdf]

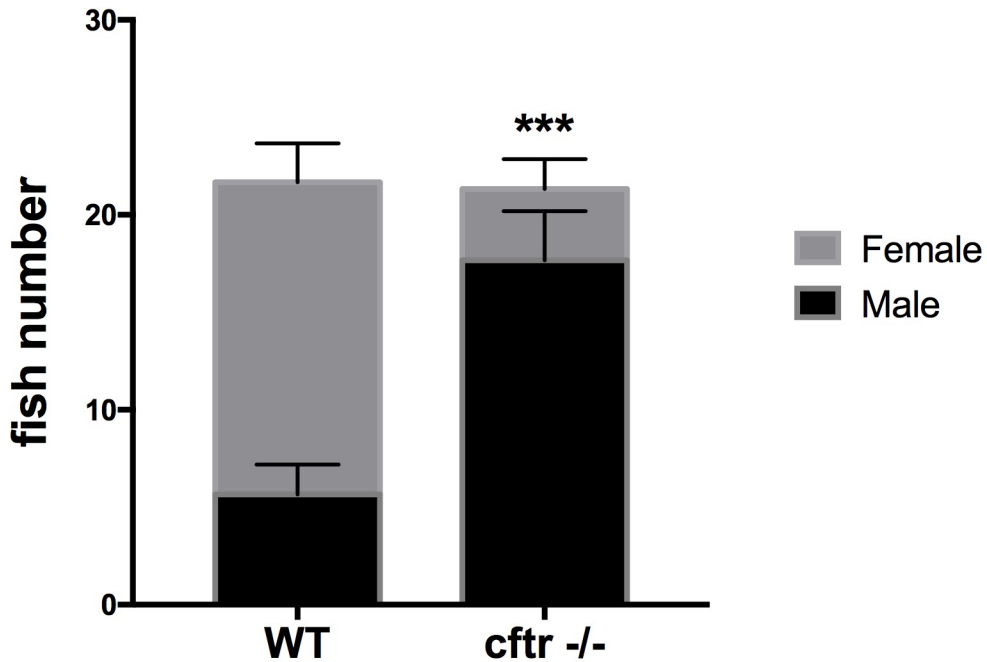

Supplement: Supporting Figure 2 [file rep-156-261-s002.pdf]
