# Supplementary material for: CFTR is required for the migration of primordial germ cells during zebrafish early embryogenesis
Source: Reproduction. 2018 Jun 21;156(3):261–8. doi: 10.1530/REP-17-0681 (PMC6106808; doi:10.1530/REP-17-0681)
Supplement: Supporting Table 1 [file rep-156-261-t001.pdf]

Supplementary Table 1 Genotype identification of offspring embryos from mutant line related to Figure 2

Stage and Marker	WT	*cftr*+/-	*cftr*-/-
**4-cell nanos1**	22% (4/18)	61% (11/18)	17% (3/18)
**4-cell vasa**	19% (3/16)	56% (9/16)	25% (4/16)
**Dome nanos1**	24% (11/45)	47% (21/45)	29% (13/45)
**Dome vasa**	26% (11/42)	50% (21/42)	24% (10/42)
**50%-Epiboly nanos1**	23% (7/31)	52% (16/31)	26% (8/31)
**50%-Epiboly vasa**	21% (4/19)	63% (12/19)	16% (3/19)
**8-somite nanos1**	27% (11/40)	53% (21/40)	20% (8/40)
**8-somite vasa**	19% (8/42)	60% (25/42)	21% (9/42)
**Prim-5 nanos1**	27% (13/49)	51% (25/49)	22% (11/49)
**Prim-5 vasa**	28% (10/36)	50% (18/36)	22% (8/36)
