# Supplementary material for: CFTR is required for the migration of primordial germ cells during zebrafish early embryogenesis
Source: Reproduction. 2018 Jun 21;156(3):261–8. doi: 10.1530/REP-17-0681 (PMC6106808; doi:10.1530/REP-17-0681)
Supplement: Supporting Table 2 [file rep-156-261-t002.pdf]

Supplementary Table 2 Genotype identification of offspring embryos from mutant line related to Figure 4

Marker and Treatment	WT	*cftr*+/-	*cftr*-/-
**nanos1-Offspring from mutant line**	25% (6/24)	54% (13/24)	21% (5/24)
**nanos1-Offspring from mutant line + WT mRNA**	27% (10/37)	49% (18/37)	24% (9/37)
**nanos1-Offspring from mutant line + ΔF508 mRNA**	27% (7/26)	50% (13/26)	23% (6/26)
**nanos1-Offspring from mutant line + G551D mRNA**	26% (11/42)	46% (16/42)	28% (12/42)
**vasa-Offspring from mutant line**	24% (7/29)	48% (14/29)	28% (8/29)
**vasa-Offspring from mutant line + WT mRNA**	24% (10/42)	55% (33/42)	21% (9/42)
**vasa-Offspring from mutant line + ΔF508 mRNA**	26% (8/31)	51% (16/31)	23% (7/31)
**vasa-Offspring from mutant line + G551D mRNA**	25% (9/36)	53% (19/36)	22% (8/36)
