# Supplementary material for: CFTR is required for the migration of primordial germ cells during zebrafish early embryogenesis
Source: Reproduction. 2018 Jun 21;156(3):261–8. doi: 10.1530/REP-17-0681 (PMC6106808; doi:10.1530/REP-17-0681)
Supplement: Supporting Table 3 [file rep-156-261-t003.pdf]

Supplementary Table 3 Genotype identification of offspring embryos from mutant line related to Figure 5D.

Stage and Marker	WT	*cftr*+/-	*cftr*-/-
**50%-Epiboly ca15b**	31% (9/29)	45% (13/29)	24% (7/29)
